# Evolving Practice in Management of Pelvic-Acetabular Trauma: COVID-19 Experience From a Tertiary Referral Centre in the UK

**DOI:** 10.7759/cureus.18778

**Published:** 2021-10-14

**Authors:** Muhammad Usman, Muhammad Yasir Tarar, Ko Ko Zayar Toe, Mohammad Iqbal, Vijaya Kempanna, Inder Gill

**Affiliations:** 1 Trauma and Orthopaedics, Salford Royal NHS Foundation Trust, Manchester, GBR

**Keywords:** orthopaedics trauma, reconstruction pelvic fractures, covid 19, acetabular fractures, pelvic ring injury, pelvi-acetabulum

## Abstract

Introduction

The United Kingdom was one of the hardest-hit countries during the COVID-19 Pandemic. The UK government announced three national lockdowns to control the spread of the coronavirus and prevent the NHS from getting overburdened with COVID-19 related attendances. Two of the most significant peaks in terms of COVID-19 related hospitalizations and COVID-19 related deaths were in Summer 2020 (corresponding to lockdown 1, which was in effect from 26th March to 26th May 2020) and early 2021 (corresponding to lockdown 3, which was in effect 6th January to 8th March 2021).

During this time, a significant proportion of NHS resources was being diverted towards the treatment of COVID-19 patients. Measures were being taken to prevent unnecessary hospitalizations and reduce patient contact. These included but were not limited to measures to reduce attendances to Emergency departments, introducing telemedicine clinics, and pausing elective services.

Our hospital is a Major Trauma Centre providing Tertiary Pelvic trauma service to the Greater Manchester area and the North West of England. We conducted this retrospective comparative study to compare the trends in presentation and Management of Pelvic trauma and identify trends in how these changed throughout the pandemic. We want to share these insights with our readers.

Methodology

We conducted a retrospective comparative study by comparing two cohorts of patients, patients presenting to the Pelvic Trauma service during Lockdown 1 and Lockdown 3 in the UK, named Group A and Group B, respectively. Data on patient demographics, injuries, and their management was identified from the Electronic Patient Record System. The data analysis was carried out with the aid of Stata/IC version 16.1. using descriptive Statistics.

Results

Group A contained 19 patients, with a mean age of 66.9 years. Group B contained 23 patients with a mean age of 67.4 years. There was no statistically significant difference in these patients' population demographics, injury patterns, and management (operative vs conservative). However, there was an absolute reduction in the complication rate from Group A to Group B of 17.2% (26.3% vs 9.1%). The higher complication rate during Lockdown 1 can be explained by conservatively managing Pelvic and Acetabular Fractures that would have been eligible for fixation, had COVID-19 not been a factor.

Conclusions

Within the limitations of our study, it appears that operatively managing a carefully selected cohort of acute Pelvic Trauma patients with proper precautions was safe and effective. It is unclear whether there was an added benefit to having a higher threshold to operate and adopting the watch-and-wait policy in Lockdown 1. We recommend continuing to follow the current evidence and fix these fractures early.

## Introduction

COVID-19 has been the cause of a significant number of deaths worldwide. As a result, a Global emergency was declared by WHO, and this outbreak was labelled a pandemic [1]. At the time of writing, United Kingdom was, and continues to be, one of the Top 10 countries affected globally concerning a total number of COVID-19 cases and deaths [2].

At the start of the pandemic, various Lockdown restrictions were put into place, reducing person-to-person contact to control the spread of COVID-19 and prevent the NHS from getting overwhelmed with the burden of COVID-19 related attendances and admissions [3]. Radical infrastructure changes such as halting elective services across the NHS, repurposing operating theatres to serve as Intensive care units, utilization of private facilities, and establishment of specific COVID-19 hospitals came into effect quite rapidly [4]. This unique situation meant the clinicians had to deviate from the established standards of care in many cases and make rational decisions based on clinical judgement due to scarcity of evidence and formal guidance: the NHS, Academy of Royal colleges and General Medical Council in the UK [5].

The first Lockdown was announced on 26th March 2020 [3]. Since then, a total of 3 lockdowns have been in effect nationally, and various levels of restrictions have been enforced locally depending on the number of new cases and deaths in those areas [3]. These lockdowns coincide with the disease burden regarding new cases diagnosed and COVID related deaths [6,7]. 

Traumatic injury patterns are linked to population behaviours, and COVID-19 Pandemic has changed the population behaviours significantly [8]. Whilst the patient demographics remain similar, there has been a reduction in the volume of traumatic adult and paediatric injuries [8-10]. The severity of these injuries has gone up on average, and a higher proportion of patients requiring surgery has been described [8,10].

There is significant focus on the recently published literature on how surgical practice, including Orthopaedic Surgery, has evolved across the globe due to the pandemic [11,12]. However, usually, the authors study these practices and compare them with Pre-COVID times, which we believe is of incredible value in its own right. However, COVID-19 Pandemic was a rapidly evolving situation where service provision was often without clear guidance and usually based on individual clinician preferences or consensus between clinicians in the form of Multidisciplinary meetings (MDT). Theoretically, this would have lead to variations in practice as the pandemic evolved. Our practice noted the change from a more conservative approach towards managing eligible injuries during Lockdown 1 towards a more liberal approach for offering fixation for eligible injuries during Lockdown 3. This hypothesis led us to analyze our practice of management of Pelvic and Acetabular trauma in the adult population, to study how our management changed, and what lessons we could learn and share with the world. 

For the reasons stated above, we conducted this retrospective comparative Cohort study where we analyzed data on patient and injury characteristics across two separate lockdowns at the beginning and towards the end of the COVID-19 wave in the UK. In theory, these two study groups would have been perfectly suited to analyze the differences in variables under study across the beginning and towards the later stages of the pandemic in the UK. 

## Materials and methods

We conducted a retrospective comparative cohort study of all the patients referred to the Tertiary Pelvic and Acetabular service during 2 National Lockdowns, 10 months apart.

Group A was all patients referred and managed by the Pelvic Trauma service during 60 days in Lockdown 1. Group B was a cohort of all the patients referred and managed during 60 days in Lockdown 3. These two cohorts of patients were approximately 10 months apart and were placed towards the pandemic's start and end, respectively. 

Patient demographics, mechanism and types of injury, management and COVID status of these patients from both the Cohorts were extracted from EPR (electronic Patient records system) and charted on an Excel Sheet. Injury types were classified anatomically based on CT scans with Multiplanar reconstruction. All Road Traffic Accidents (RTAs) and falls from height > 1m were regarded as high energy injuries, whereas falls from standing height or < 1m height were regarded as Low energy injuries. Decisions on the management of these injuries were made based on MDT consensus, expertise available, and the individual surgeons preference at the time of injury, taking into account patient preference and the risks related to COVID-19 infection. During Lockdown 1, we had a more conservative approach towards patient management and many injuries that would have been routinely managed operatively were given a trial of conservative management. When patients were managed conservatively, they were advised full weight-bearing and analgesia, whilst the parent medical teams were advised to report any fracture-related complications including pain that was refractory to medical therapy. During Lockdown 3, all patients qualifying for operative fixation had their fractures fixed early except for one patient. 

The data analysis was carried out with the aid of Stata/IC version 16.1. It was carried out using descriptive statistics, Fisher exact test and independent-sample t-test. An independent sample t-test was used to compare the mean age between the patient groups. Fisher exact test was used to determine a correlation between the Lockdown periods and other variables and determine a correlation between Complications and different variables. Bivariate logistic regression analysis was used to determine the correlation between Lockdown periods and the development of complications. A p-value of less than 0.05 was considered statistically significant. 

## Results

A total of 19 patients were included in Cohort A (patients presenting during Lockdown 1), with a mean age of 67.3, of which 68.4% were males. Cohort B (patients presenting during Lockdown 3) contained 23 patients with a mean age of 67.4, of which 47.8 % were males. Overall, we did not see a statistically significant change in population demographics across both the Cohorts. Mechanisms and types of injury also remained similar across both cohorts. Population and injury demographics are outlined in Table 1. 

**Table 1:** Summary of patient and injury characteristics in both Cohorts * 1 patient lost to follow-up RTA- Renal tubular acidosis

Variable	Group A (N=19)	Group B (N=23)	Total (N=42)	p-value
Gender				
Male	68.4%	47.8%	57.1%	0.1
Female	31.6%	52.2%	42.9%	
Mean age	66.9 ± 20.3	67.4 ± 21.9	67.2 ± 20.9	0.9
Type of injury				
High Energy	52.6%	43.5%	47.6%	0.4
Low energy	47.4%	56.5%	52.4%	
Mechanism of injury				
Simple fall	47.4%	56.5%	52.4%	0.9
Fall from height	26.3%	17.4%	21.4%	
RTA	26.3%	26.1%	26.2%	
Injury classification				
Acetabular Fx	52.6%	43.5%	47.6%	2.2
Isolated Pelvic injury	10.5%	8.7%	9.5%	
Pelvic Ring Injuries	36.8%	43.5%	40.5%	
Combined Injury	0%	4.4%	2.4%	
Patient Management				
Managed Locally	73.7%	60.9%	66.7%	0.4
Presented to our Hospital	5.3%	21.7%	14.3%	
Presented Locally & transferred to our Hospital	21%	17.4%	19%	
Conservative vs Operative				
Conservative	78.9%	78.3%	80.1%	0.5
Operative	21%	21.7%	19.0%	
Complications *				
Yes	26.3%	9.1%	17.1%	0.1
No	73.7%	90.9%	82.9%	

Acetabular fractures made up nearly half of each cohort (52.6% in cohort A; 43.5% in cohort B). The least prevalent injuries were combined acetabular and pelvic ring injuries ( 0% in Group A vs 4.4% in Group B). Across both cohorts, 66.7% of the patients were managed in their local hospitals with our advice, whereas 33.4% (26.3% in Group A; 39.1% in Group B) either presented directly or were transferred across for assessment and/ or operative intervention. All patients managed at our Trauma centre had COVID-19 PCR tests at various points during their management, and none of them had a positive COVID-19 test result. COVID-19 data for patients managed elsewhere was not available with certainty and thus not recorded. 

**Figure 1:**
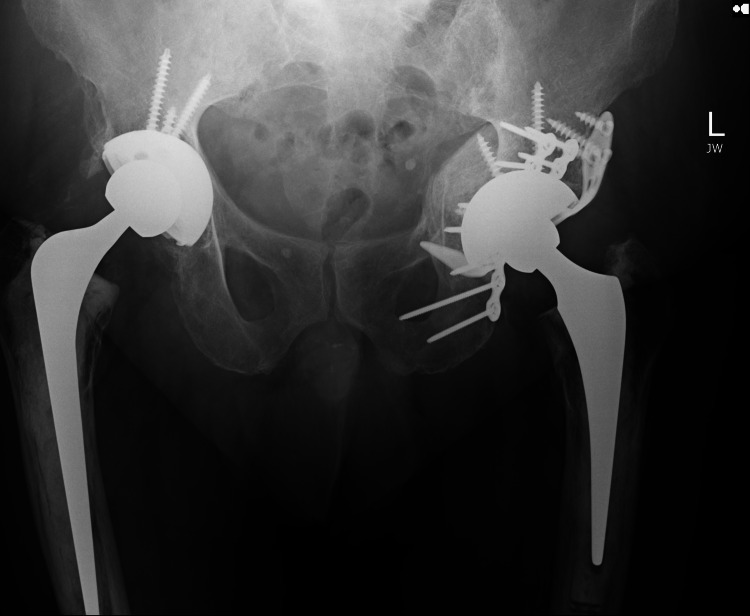
Left Total Hip replacement in a patient 6 months after Acetabular Fracture using a Cup-Cage construct* * Previous Right Total Hip replacement in situ

**Figure 2:**
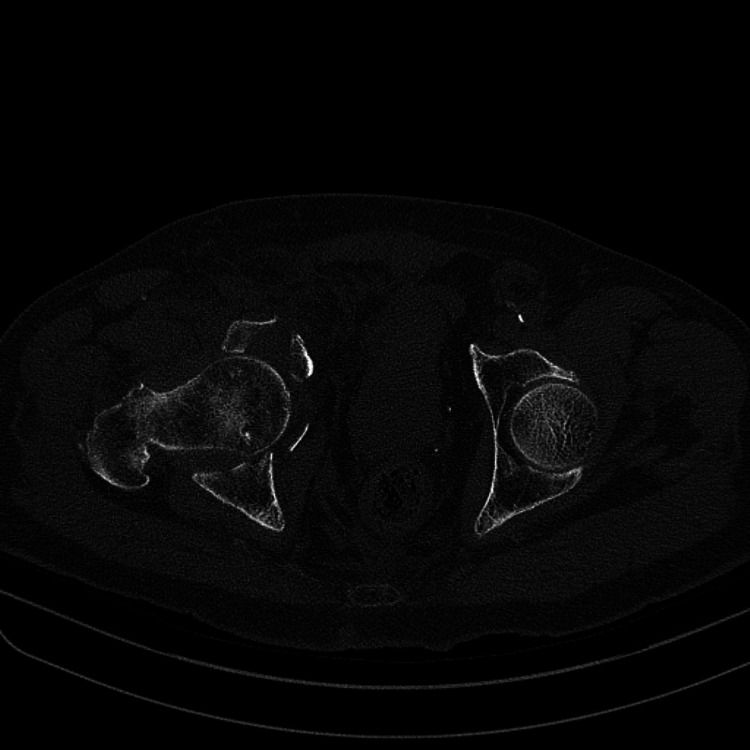
Right Acetabular fracture with Medial migration of the Femoral Head (Axial view)

**Figure 3:**
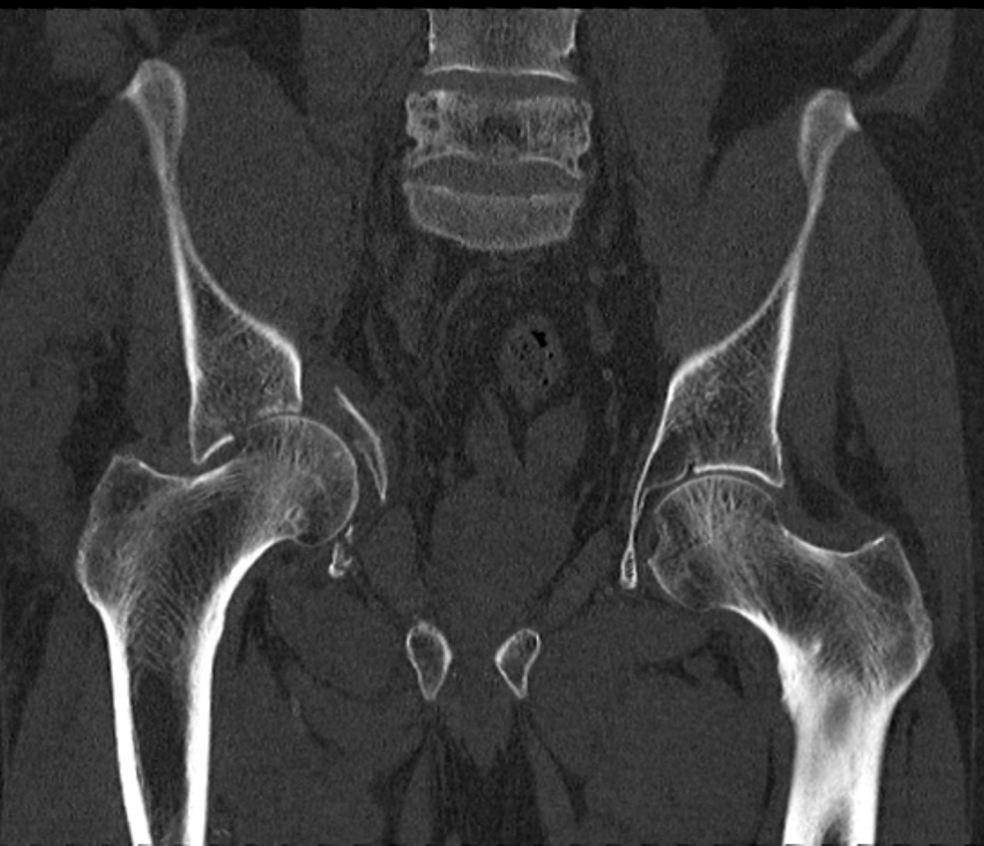
Right Acetabular fracture with Medial migration of the Femoral Head (Coronal view)

**Figure 4:**
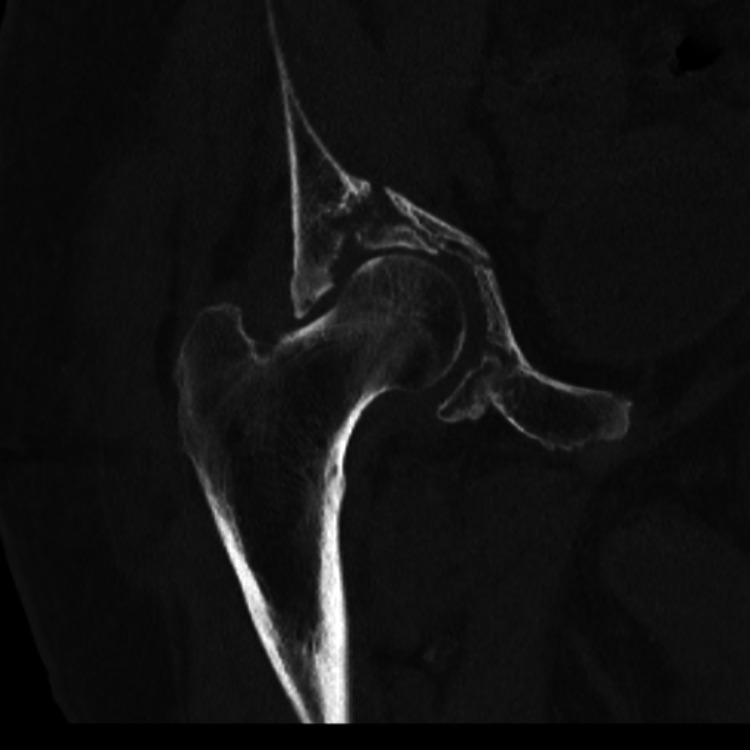
CT scan demonstrating Right Acetabular Fracture Non-Union 5 months post-injury (coronal view)

**Figure 5:**
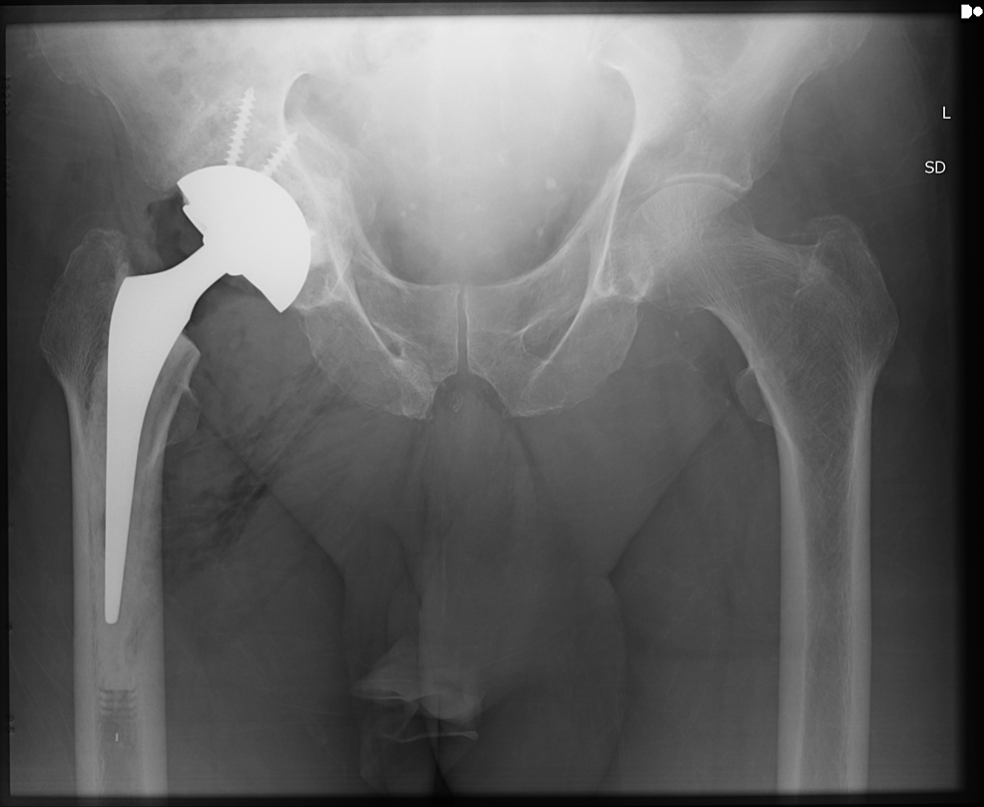
Right Total Hip replacement with Supra-Acetabular screws and Bone Grafting, for Acetabular Fracture Non-Union

26.3% of the patients managed by the Pelvic Trauma service during Lockdown 1 developed fracture-related complications. This is in contrast to the patients managed during Lockdown 3, 9.1% of which developed complications [Table 1]. Patient demographics, injury morphology, complications, and management are outlined in tables 2, 3 respectively. One patient in Group B died from medical causes unrelated to the fracture and was considered lost to follow up. Overall, 17% of the patients across both Cohorts developed complications. 71.5% of these complications happened during lockdown 1, and 28.5% happened in lockdown 3. 60% of Group A complications were in patients deemed eligible for operative fixation, had COVID-19 not been a factor, but were managed conservatively. These patients formed 20% of the conservatively managed patients in Group A and failed this trial of conservative management. These patients had ongoing chronic pain and poor quality of life, and follow up imaging demonstrated ongoing non-union, further migration of the head and fracture displacement. All of these patients underwent complex total hip replacements after the lockdown was over. Most of these patients needed bone grafting, some of them needed augments and a Cup-cage construct secondary to significant Protrusio Acetabuli and bone loss [Figure 1]. One patient developed a similar complication in Group B. This patient had an acetabular fracture with medial migration of the femoral head, was trialled on conservative management based on individual surgeon's preference, and later received a delayed total hip replacement [Table 3, Figures 2-5]. With this exception, all patients who qualified for fixation had their fractures fixed early. 

**Table 2:** Complications in Group A M = Male; F= Female; OA= Osteoarthritis; ORIF- Open reduction internal fixation

Patient characteristics	Injury pattern	Management in acute phase	Choice of Procedure	Complication	Management of Complication
61M	Acetabular Fracture	Conservative	N/A	Ongoing Pain, Secondary OA, Non-Union of Fracture	Acetabular Fracture Fixation, Bone grafting and Complex Primary Total Hip replacement
81M	Acetabular Fracture	Conservative	N/A	Chronic Pain, Non-Union and secondary OA	ORIF Acetabulum, Bone Grafting and Complex Primary Total Hip replacement using Cup-Cage construct
74M	Acetabular Fracture	Conservative	N/A	Non-Union and Secondary OA	Fixation of Acetabular Non-union and Primary Total Hip replacement
26F	Acetabular Fracture	Operative	ORIF Acetabulum; Percutaneous LC2 screws and Supra-acetabular screws	Pulmonary Embolism	Medical Management with Anti-coagulation
84M	Pelvic Ring injury	Operative	Illiosacral Screws	Displacement of Vertical Shear component; Asymptomatic Mal-union	Fracture union with Conservative management. Patient Asymptomatic

**Table 3:** Complications in Group B * Figures 2-5

Patient Characteristics	Injury pattern	Management in acute phase	Choice of Procedure	Complication	Management of Complication
68M	Pelvic Ring injury	Operative	Bilateral Illiosacral screws and Pelvic Infix	Asymptomatic Malunion/ Fibrous union of Pubic Ramus, Irritation from Pelvic Infix	Symptoms resolved with Removal of Pelvic infix
84M	Acetabular Fracture*	Conservative	N/A	Chronic Hip pain, Non-union and secondary OA*	Complex Total Hip replacement with Supra-acetabular screws and Bone graft*

 

 

**Figure 6:**
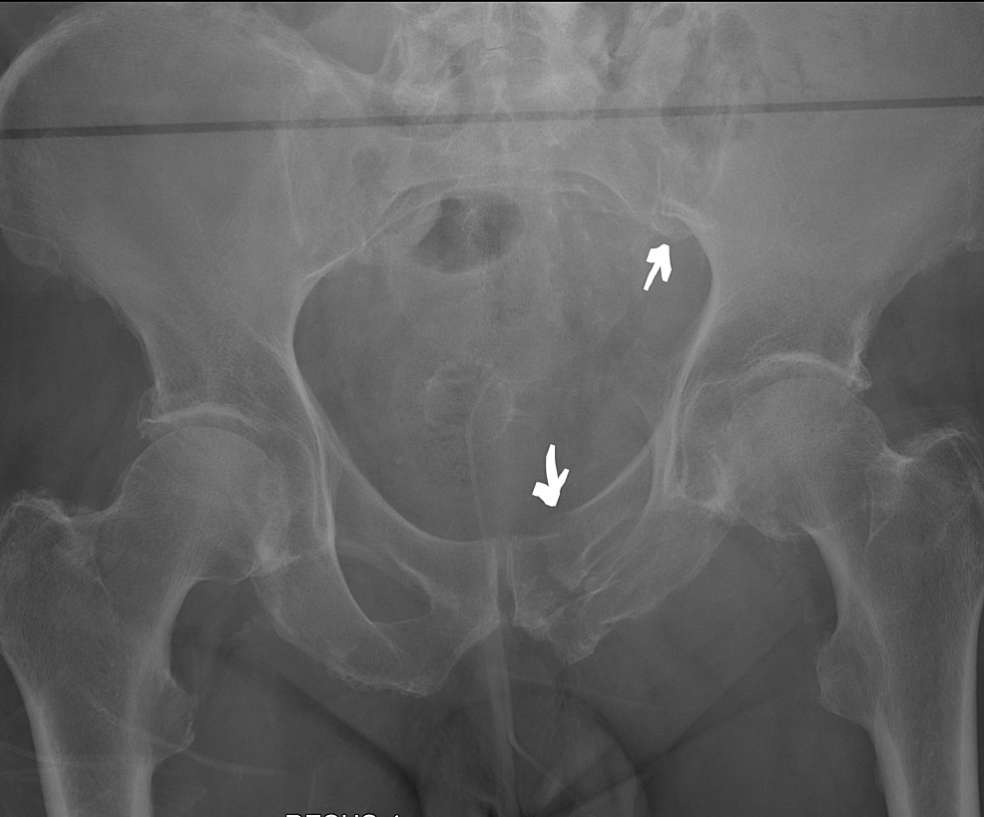
AP Radiograph demonstrating Pelvic Ring injury with Subtle Superior displacement of Left Hemipelvis* (Vertical Shear Fracture) *Arrows indicating points of Fracture and superior displacement of Left Hemipelvis

**Figure 7:**
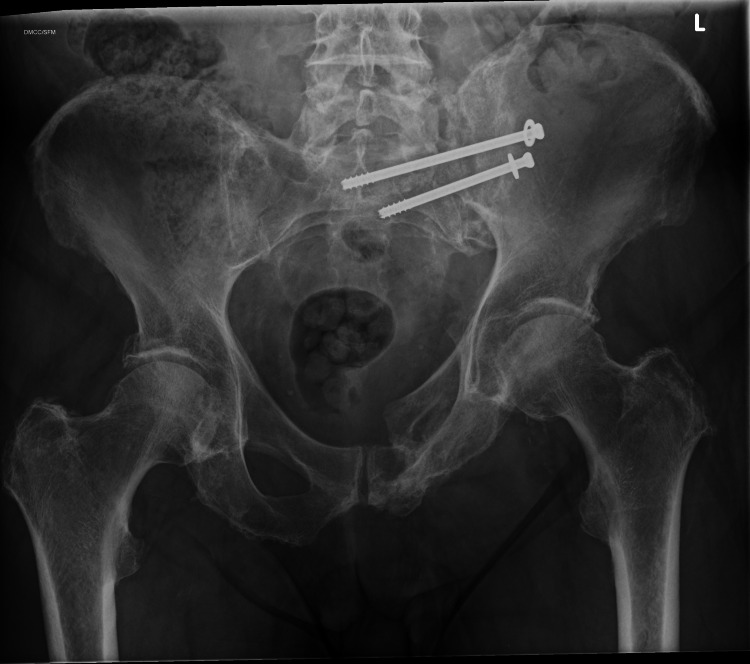
Further superior displacement of Left Hemipelvis after left sided Illiosacral screws

One patient from Cohort A with an LC2 type fracture and a vertical shear component was managed with S1 and S2 Illiosacral screws [Table 2]. This patient had a subtle displacement of the vertical shear component on follow up; however, the patient remains asymptomatic, and the fracture is healed on further follow up [Figures 6, 7].

When analyzing complications cumulatively across both cohorts, no statistically significant difference could be demonstrated between the development of complications and Lockdown durations, patient's age, or injury morphology [Table 4]. Bivariate logistic regression analysis of complications compared with Lockdowns demonstrated that patients were 1.9 times more likely to develop complications during lockdown 1 than Lockdown 3. However, the p-value was 0.2, and this correlation was thus considered not significant.

**Table 4:** Analysis of Complications and other variables * 1 patient during Lockdown 3 lost to followup

Variable	Complications		p-value
	Yes N=7	No N=34*	
Period of Lockdown			
Group A	71.4%	41.17%	0.1
Group B	28.5%	58.82%	
Mechanism of injury			
Simple fall	28.5%	55.9%	0.2
Fall from height	14.2%	23.52%	
RTA	57.1%	20.6%	
Mechanism of Injury			
High energy	71.4%	44.1%	0.2
Low energy	28.5%	55.8%	
Patient management type			
Conservative	71.4%	79.4%	0.4
Operative	28.5%	17.6%	
Mean age	68.1 ±20.5	68.9 ± 21.5	0.9
Injury classification			
Acetabular Fractures	71.5%	44.1%	0.8
Isolated Pelvic injury	0%	11.8%	
Pelvic Ring Injuries	28.5%	41.2%	
Combined Injury	0%	2.9%	

All the managed or presented to our hospital patients were tested for COVID-19, and none of them had a positive result. All the operations were done under full personal protective equipment (PPE) following national and local PPE policy guidance at the time of surgery. None of the patients had any COVID-19 related complications following surgery.

## Discussion

Pelvic and acetabular fractures are a major cause of disease and operative burden on the NHS, and this burden is increasing every year [13]. They are associated with increased mortality risk, extended hospital stay, and ICU admission likelihood [14,15]. Distribution is usually bi-modal, with younger patients suffering from high energy injuries and low energy falls being the predominant mechanism of injury in elderly patients [16]. In-hospital mortality rates for elderly patients sustaining Pelvic Fractures have been reported to be similar to those of the Neck of Femur Fractures [17]. In their study of Pelvic and Acetabular Trauma in Ireland during the COVID-19 pandemic. Mohan et al. [18] report that the burden of Pelvic-Acetabular trauma during the COVID-19 pandemic has similar to pre-pandemic levels in terms of patient and injury demographics and the number of patients requiring surgical intervention. While smaller number o patients present to the hospital with traumatic injuries, these injuries' severity has increased on average [8-10]. These changes would be difficult to generalize based on the variability of restrictions, socioeconomic factors and structure of healthcare services across various geographical regions of the world. Our findings suggest that these factors stayed consistent throughout the lockdowns when comparing population demographics and injury patterns in adult patients. 

Initial management and stabilization of these injuries follow the Advanced trauma life support (ATLS) guidelines, and application of a Pelvic binder in the case of suspected pelvic fractures pre-hospital is recommended by the British Orthopaedic Association (BOA) [19]. BOA also recommends consideration of External Fixation in mechanically unstable Pelvic Fractures and considering early Pelvic ring reconstruction within 72 hours if the patient is physiologically stable [19]. The decision to operate and the surgical technique used depends on the type of injury, patient-specific factors and the surgeon's preference [16,20].

In their systematic review on the optimal timing of Pelvic fixation, Katsoulis et al. [21] concluded that early fixation of Pelvic and Acetabular fractures was recommended to achieve optimal outcomes. Delayed Pelvic fixation was associated with a higher risk of complications such as inability to achieve anatomical reduction, involvement of more extensive approaches, the difficulty of operative treatment, higher failure rate, and an overall reduction in optimal outcomes. [21-24]. The definition of 'early fixation' in literature is, however, poorly characterized [21]. 

We aim to fix all pelvic and acetabular fractures that merit fixation as early as possible unless the patient is hemodynamically unstable and unable to tolerate the physiological hit from major surgery. Choice of management and the choice of specific procedure depends on MDT consensus, operating surgeons' preference and logistical barriers to fixation, e.g. availability of theatre lists, clinical priority and technical expertise of the available Orthopedic surgeons at the time. During the COVID-19 pandemic, there was an additional perceived risk associated with nosocomial COVID-19 infection. It is worth noting that reliable data during the early pandemic about infection rates in surgical patients was missing, and the decisions had to be based on perceived risk. As evidence started to surface, it confirmed the fears of clinicians with postoperative mortality rates in COVID-19 patients reported to be between 20% and 23.8% and the need for ICU admissions up to 64% in case of patients undergoing major surgery [25,26]. A correlation with increased perioperative mortality was also reported with Major vs Minor surgeries, age> 70 years, and higher ASA grades [25]. 

BOAST (British Orthopedics Association standards for Trauma and Orthopaedics) and Royal College of Surgeons, England, guided surgical management of patients during COVID-19 [27,28]. This included considering non-operative management of injuries, performing surgeries as day cases and minimizing the length of stay in the hospitals. Specific guidance on Pelvic-acetabular trauma, however, was scarce. Our departmental policy during the pandemic recommended conservative management of all acetabular fractures and all Pelvic Ring injuries without haemodynamic instability. Management of the Pelvic Trauma patients in our hospital was based on MDT discussion after considering the guidance above, focusing on conservative vs surgical management and choice of surgical procedures to minimize operating time wherever possible. MDT decisions vary from patient to patient depending on many factors such as ASA grade, pre-injury functional baseline and fracture configuration, to name a few. However, the principles are based on the best available evidence and usually follow guidance from British Orthopaedic Association Standards for Trauma (BOASTs), as discussed above. All MDT decisions during the pandemic were made within the confines of patient safety as thought appropriate at the time. We believe that Human factors in clinical decision making do not get talked about enough in the context of COVID-19; however, risks of clinicians acquiring COVID-19 infections, constantly changing PPE guidance, supply shortages in PPE and the resulting apprehension among clinicians would have also influenced the clinical decisions made.

These factors made decision making in unstable Pelvic and Acetabular fractures that would have otherwise been operated on and fixed in the acute phase a challenge. A significant proportion of patients who were a candidate for surgery had COVID-19 not been a factor, failed a trial of conservative management. It is thus arguable that the benefit of treating these patients conservatively and avoiding surgery was offset by a poorer outcome and more complicated operations later, in the form of Total Hip arthroplasties, bone grafting and fixation. Although all of these patients made an uncomplicated recovery after their delayed operation at the time of writing, we suspect these patients are more prone to complications related to technical failure. Subsequent revision of their total hip arthroplasty (THAs), if required, will be more complex, with associated costs to patients and the healthcare system.

Our practise evolved as the pandemic progressed after observing this trend. During the 3rd lockdown, we had a lower threshold to offer operative fixation for eligible patients, and our study reflects that. Except for one patient who was trialled on conservative management despite being a surgical candidate, all patients who qualified for fixation had their fractures fixed early. Another important thing to note is the influence of the pandemic on the choice of procedures performed. One patient with an LC2 type fracture and a vertical shear component within our Cohort A was managed with S1 and S2 Illiosacral screws. On reflection, this patient could have been more optimally managed with Trans-sacral screws and a Pelvic Infix; however, the operative time was a consideration, and the decision to perform Illiosacral screws was made. Surgical procedures during lockdown 3, however, demonstrate less of an influence by COVID-19. We believe that this was multifactorial due to the rollout of a vaccination programme, the evolution of our understanding around COVID-19, and lessons learned from the management of patients during lockdown 1.

Limitations

We did not follow up on all of the conservatively managed patients within our two cohorts, and it is possible that complications directly unrelated to the Pelvic Fractures wouldn't have been reported to us. However, we are confident in the key metric that any complications related to fracture and its management, such as ongoing pain refractory to medical therapy, Non-union, mal-union and development of pulmonary embolism/deep vein thrombosis (PE/DVT), would have been brought to our attention. In addition, we were not able to determine statistically significant correlations between lockdown periods and the development of complications due to the small sample size. This was reliant upon the volume of trauma we received from the Northwest of England during lockdown periods; we did see the volume of trauma patients dry up during the lockdown periods quite significantly, as described in the literature and discussed above. However, we felt that extending the study period beyond the lockdown periods would have invalidated the point of doing our study and skewed the findings. Objective quality of life for patients managed during the pandemic could not be reported as patients were managed at various sites, and our records to assess these scores were inadequate accurately. The retrospective nature of data collection also contributed to this limitation. Based on our hospital policy, isolated Pubic Rami Fractures that are undisplaced or minimally displaced are not brought to the attention of the Orthopaedic on-call service or the Pelvic Trauma service. It is thus possible that our cohort underestimates the incidence of Low energy injuries during the two lockdowns.

## Conclusions

Injury patterns and patient demographics appear to be the same across the COVID-19 peaks in the relevant literature and our study. We did, however, attempt to manage patients eligible for operative fixation, conservatively during lockdown 1. This lead to these patients progressing to Non-union or malunion and chronic pain with resulting limitations in daily activities. Within the limitations of our study, it appears that all patients who qualified for surgical fixation during lockdown 1 should have been offered a surgical fixation. Doing so whilst following the PPE guidance and taking proper safety precautions would have been safe and effective. This would have allowed early rehabilitation, avoided delayed operations with greater technical complexity, and carried a higher risk of complications down the line. We advocate early operative intervention for patients with unstable Pelvic-Acetabular injuries. We wanted to share our experience with the readers, hoping that it will inform their practice in the future as it did for us as the pandemic evolved.
